# Studies on Children With Developmental Coordination Disorder in the Past 20 Years: A Bibliometric Analysis *via* CiteSpace

**DOI:** 10.3389/fpsyt.2021.776883

**Published:** 2021-12-06

**Authors:** Mei-Qin Wu, De-Qing Wu, Chun-Ping Hu, Lai-Sang Iao

**Affiliations:** ^1^Shanghai Key Laboratory of Maternal Fetal Medicine, Department of Women's and Children's Health Care, Shanghai First Maternity and Infant Hospital, School of Medicine, Tongji University, Shanghai, China; ^2^Department of Gastroenterology, Shanghai Tenth People's Hospital, Tongji University School of Medicine, Shanghai, China; ^3^School of Public Health, Shanghai Jiao Tong University School of Medicine, Shanghai, China; ^4^Department of Psychology, Nottingham Trent University, Nottingham, United Kingdom

**Keywords:** developmental coordination disorder, children, web of science, data visualization, burst detection

## Abstract

Children with developmental coordination disorder (DCD) have been commonly observed and drawn an increasing amount of attention over the past decades. The aim of the present study is to evaluate the origin, current hotspots, and research trends on children with DCD using a bibliometric tool. After searching with “children” and “developmental coordination disorder” as the “topic” and “title” words, respectively, 635 original articles with 12,559 references were obtained from the electronic databases, Web of Science Core Collection (WoSCC). CiteSpace V.5.7.R2 was used to perform the analysis. The number of publications in this field was increasing over the past two decades. John Cairney from the Department of Family Medicine, McMaster University, Canada, was found to be the most productive researcher. Meanwhile, McMaster University and Canada were the most productive research institution and country, respectively. Reference and journal co-citation analyses revealed the top landmark articles and clusters in this field. *Clumsiness* was the most strength burst keyword. Moreover, *task, meta-analysis, difficulty, adult*, and *impact* will be the active research hotspots in future. These findings provide the trends and frontiers in the field of children with DCD, and valuable information for clinicians and scientists to identify new perspectives with potential collaborators and cooperative countries.

## Introduction

Children with developmental coordination disorder (DCD) exhibit severe motor clumsiness that interferes with academic achievement and the activities of daily living (1). Symptoms of DCD occur in the early stage of development, but it is often not identified until school age, leaving missed good opportunities for early intervention (2). These children have previously been described with a variety of terms such as developmental dyspraxia, minimal brain dysfunction, perceptual-motor dysfunction, physical awkwardness, or, most commonly, the clumsy child syndrome (3, 4). In 1994, these children were collectively referred to as DCD at an international consensus meeting which held in London, Ontario (5).

In recent years, there has been growing recognition of the consequences, outcomes, and burden for children with DCD. However, after the London consensus meeting, DCD, as a unified terminology, lacks systematic research on global research trends and hotspots in this field. Evaluative bibliometrics is a field of quantitative science that has emerged as a powerful tool to evaluate research performance, which can serve to identify influential articles that have shaped medical practice and fostered new research ideas (6).

CiteSpace, one of the bibliometric analysis tools, which was invented by Dr. Chaomei Chen (School of Information Science and Technology, Drexel University, Philadelphia, PA, USA), has been widely used in other research fields (7–9). Professor Chen Chaomei, as the inventor of CiteSpace, published a visual analysis of the emerging research trends in the field of regenerative medicine, on the Expert Opinion on Biological Therapy in May 2012 (10). This study accurately predicted the winner of the 2012 Nobel Prize in physiology/medicine, the Japanese scholar Shinya Yamanaka (11, 12). This empirical study shows that CiteSpace software plays an important role in literature mining.

However, to the best of our knowledge, CiteSpace analysis has not been reported in the field of children with DCD. In this study, the literatures related to children with DCD were collected and screened to form a specific database. Then, CiteSpace was used to perform statistical calculations and further generate visualization results for different node types. The purpose of this article is to clearly visualize and explain the origin and major milestones of the research in children with DCD, after DCD was used as a unified terminology.

## Methods

### Data Source

The retrieval data for measurements and statistical analysis were screened from the Web of Science Core Collection (WoSCC), which provides citation search, giving access to multiple databases that reference cross-disciplinary research and allowing for an in-depth exploration of specialized subfields (13).

### Search Strategy

All data were obtained from WoSCC on May 10, 2021; the data retrieval strategy was as follows: (i) Topic = children and Title = developmental coordination disorder; (ii) Document type = article; (iii) Language = English; (iv) Timespan (custom year range) = 2000–2019. Full records and cited references were selected as plain text format and downloaded for further analysis.

### Analysis Tool

This study utilized CiteSpace V.5.7.R2 to analyze existing studies related to children with DCD, aiming to provide scientific and intuitive support for clinicians and researchers in this field. CiteSpace, which was created by Dr. Chaomei Chen (School of Information Science and Technology, Drexel University, Philadelphia, PA, USA) and his team in early 2004 (14), was used to perform the bibliometric analysis in this study. CiteSpace is a Java application which combines information visualization methods, bibliometrics, and data mining algorithms in an interactive visualization tool.

### Data Analysis

Two separate folders for the DCD project were created. One folder contains data files which were just downloaded. The other folder is the project folder. We did not find duplicate documents that needed to be deleted. The overall selected time span was from January 2000 to December 2019. Then, the time span was sliced into 10 parts corresponding to 10 different colors, each of which was 2 years. The node type was selected according to the type of analysis performed. The size of the circles represents the number of papers published by the country/region, institute or author. The shorter the distance between two circles, the greater the cooperation between the two countries/regions, institutes or authors. Purple rings indicate that these countries/regions, institutes or authors have greater centrality (no <0.1).

## Results

### General Data

**Figure 1:**
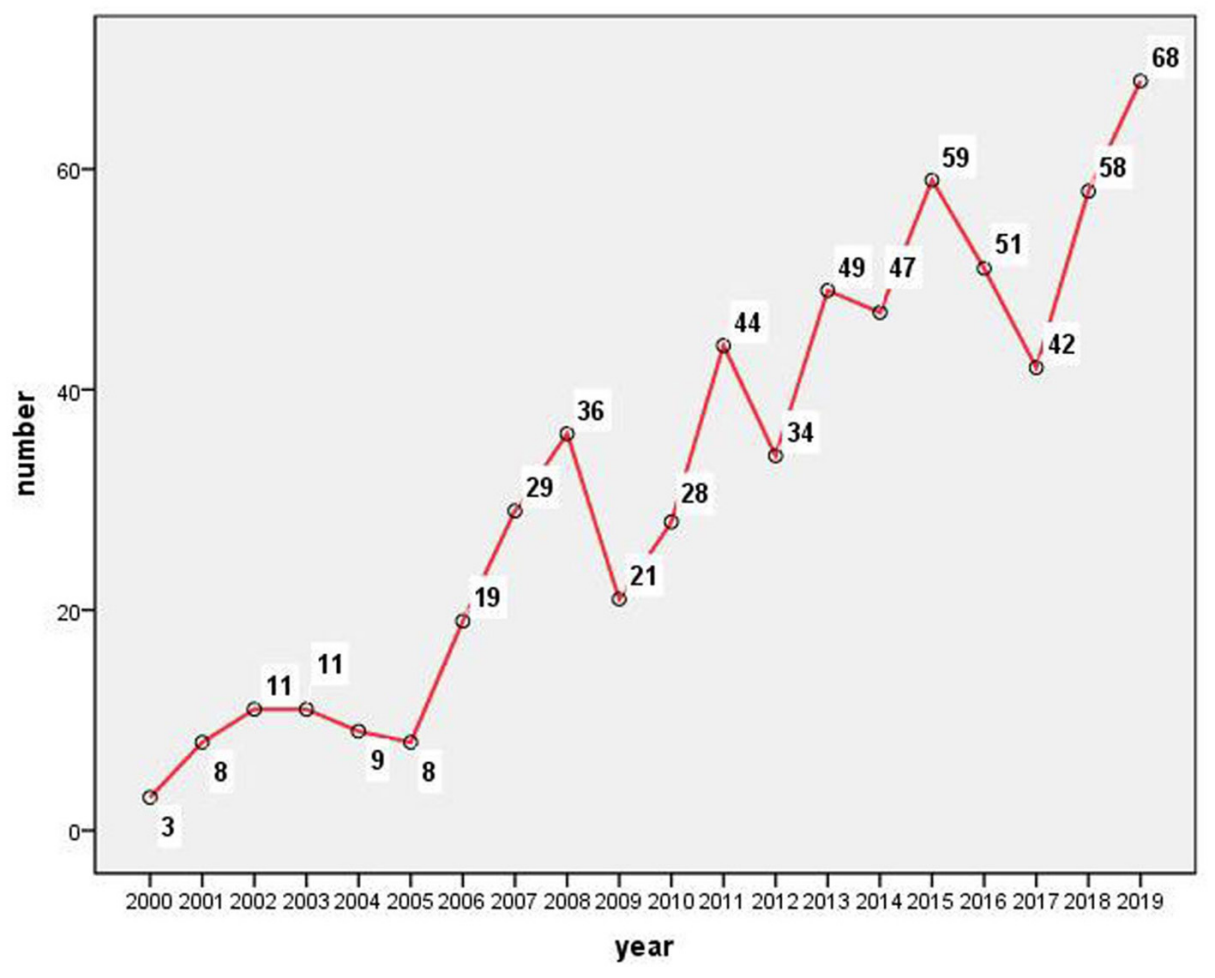
The number of children with DCD publications indexed by WoSCC, 2000–2019.

The initial search for children with DCD resulted in 635 original articles published in English between 2000 and 2019, without duplicate record. According to the publication years, although with some fluctuations, the quantity of published articles on children with DCD increased significantly over the studied period, especially from 2005 to 2008, with an average annual growth rate of 65.10% (Figure 1).

### Quantitative and Cooperation Analysis

#### Countries/Regions

**Figure 2:**
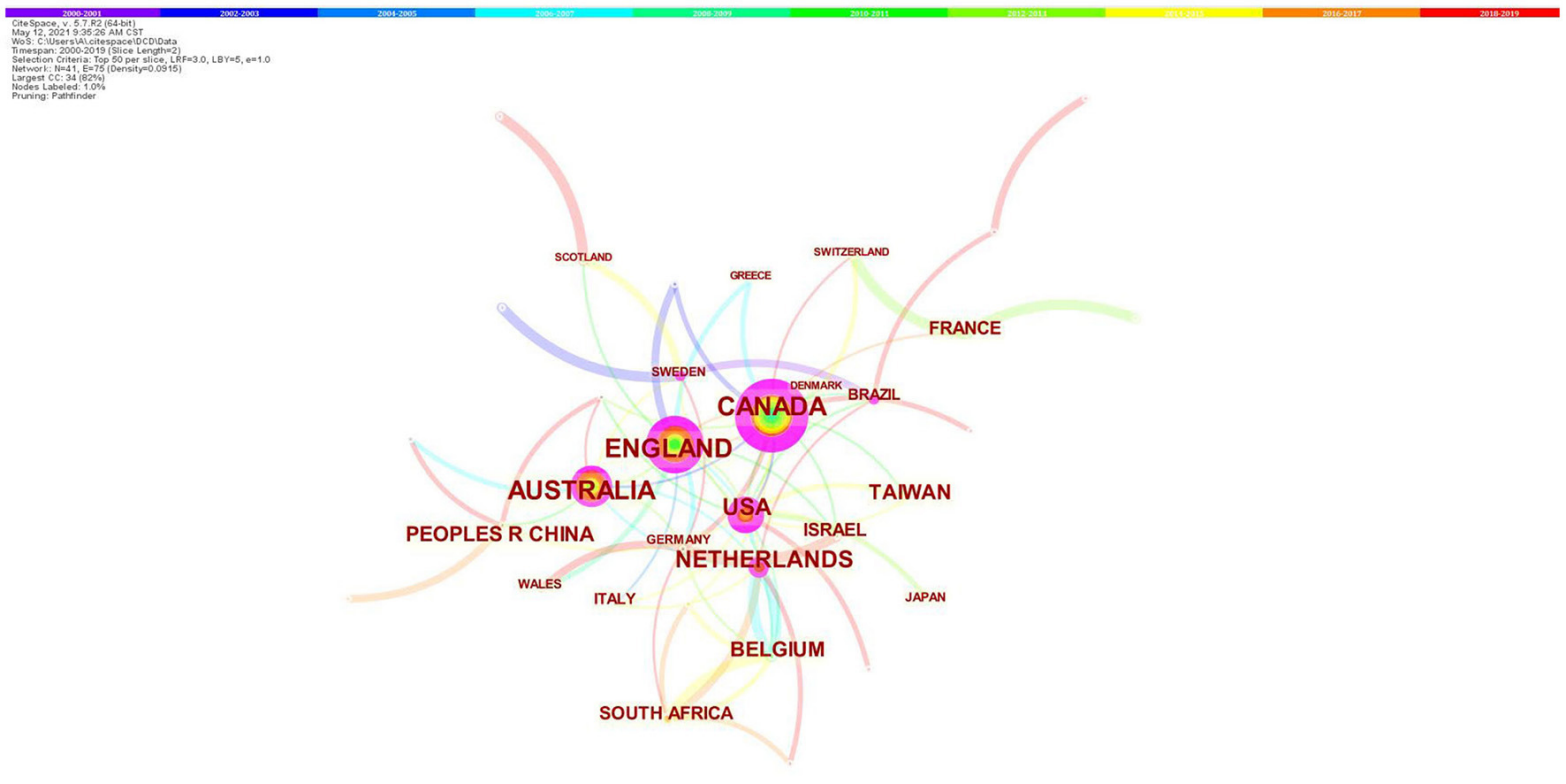
Map of countries/regions cooperative relations in research of children with DCD, 2000–2019. The bigger the circle, the more original articles the country/region published. The shorter and thicker the connection line, the closer the relationship.

Figure 2 displays the cooperation network of the productive countries/regions. The size of circles represents the number of publications of countries/regions, and the shorter distance between two circles suggests the more collaboration between individual countries/regions. The purple rings outside the circle refer to centrality. Canada (137) ranks first in the publication quantity, which is followed by England (114) and Australia (104). The top 10 prolific countries/territories in this research field are shown in Table 1, which are sorted out from the network summary table.

**Table 1:** Top 10 prolific countries/regions in research of children with DCD, 2000–2019.

**Ranking**	**Country/region**	**Frequency**	**Centrality**	**Ranking**	**Country/region**	**Frequency**	**Centrality**
1	CANADA	137	0.44	6	TAIWAN	51	0.00
2	ENGLAND	114	0.24	7	BELGIUM	36	0.04
3	AUSTRALIA	104	0.15	8	PEOPLES R CHINA	36	0.10
4	USA	73	0.37	9	SOUTH AFRICA	31	0.02
5	NETHERLANDS	72	0.13	10	FRANCE	26	0.08

#### Institutes

**Figure 3:**
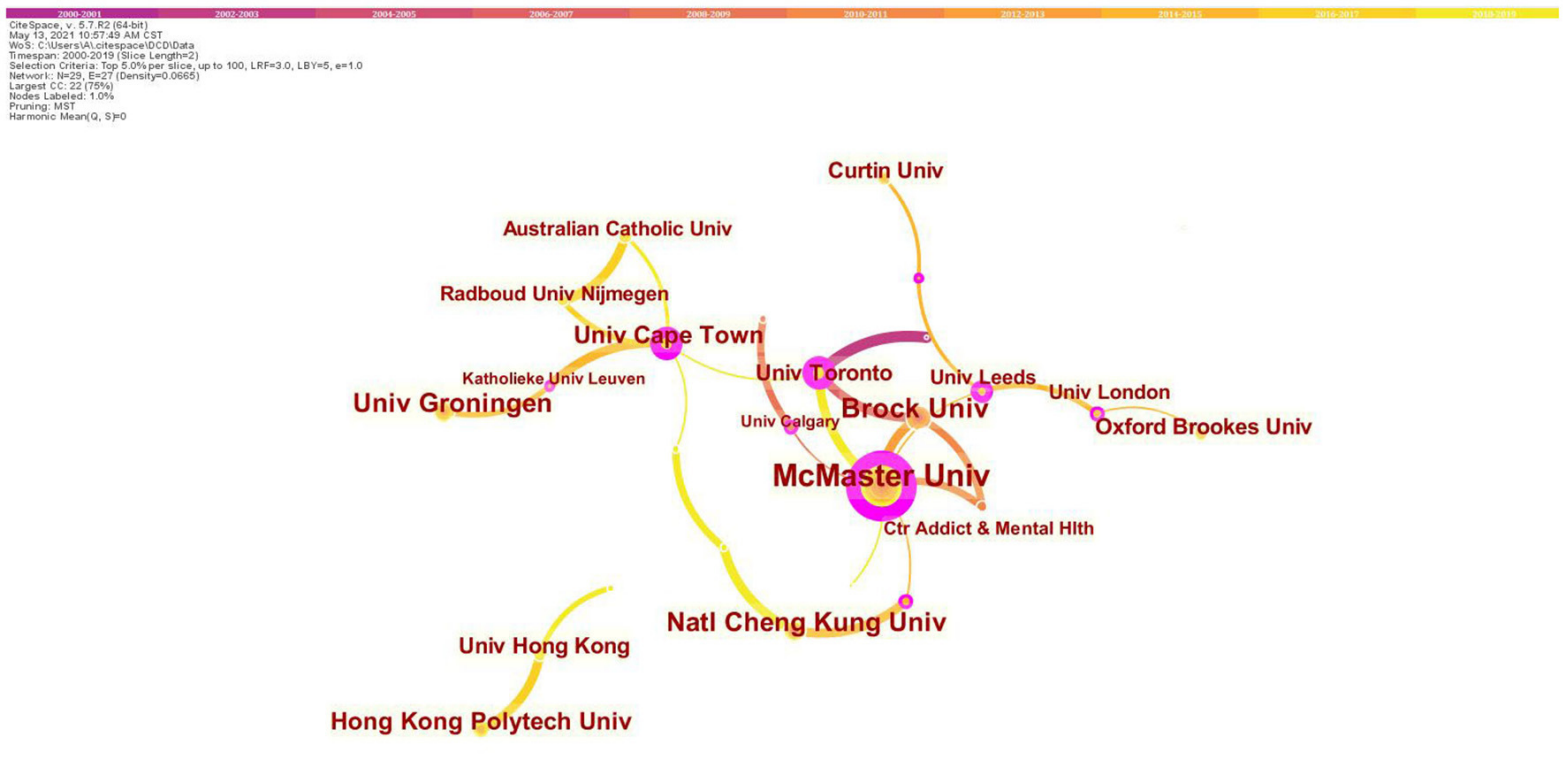
Map of institutes' cooperative relations in research of children with DCD, 2000–2019. The bigger the circle, the more original articles the institute published. The shorter and thicker the connection line, the closer the relationship.

Figure 3 exhibits the major productive co-institutes in the field of children with DCD. The Canadian McMaster University is the most productive and influential institute in this field, with a total number of 54 published articles, and the centrality of 0.74. Brock University, which ranks second in productivity, also locates in Canada. The top 10 prolific institutes in this research field are shown in Table 2.

**Table 2:** Top 10 prolific institutes in research of children with DCD, 2000–2019.

**Ranking**	**Institute**	**Frequency**	**Centrality**
1	McMaster University	54	0.74
2	Brock University	27	0.02
3	University of Groningen	24	0.00
4	Natl Cheng Kung University	22	0.07
5	University of Cape Town	20	0.46
6	Hong Kong Polytechnic University	19	0.00
7	University of Toronto	15	0.48
8	Oxford Brookes University	15	0.00
9	University of Hong Kong	14	0.01
10	Curtin University	13	0.00

#### Authors

**Figure 4:**
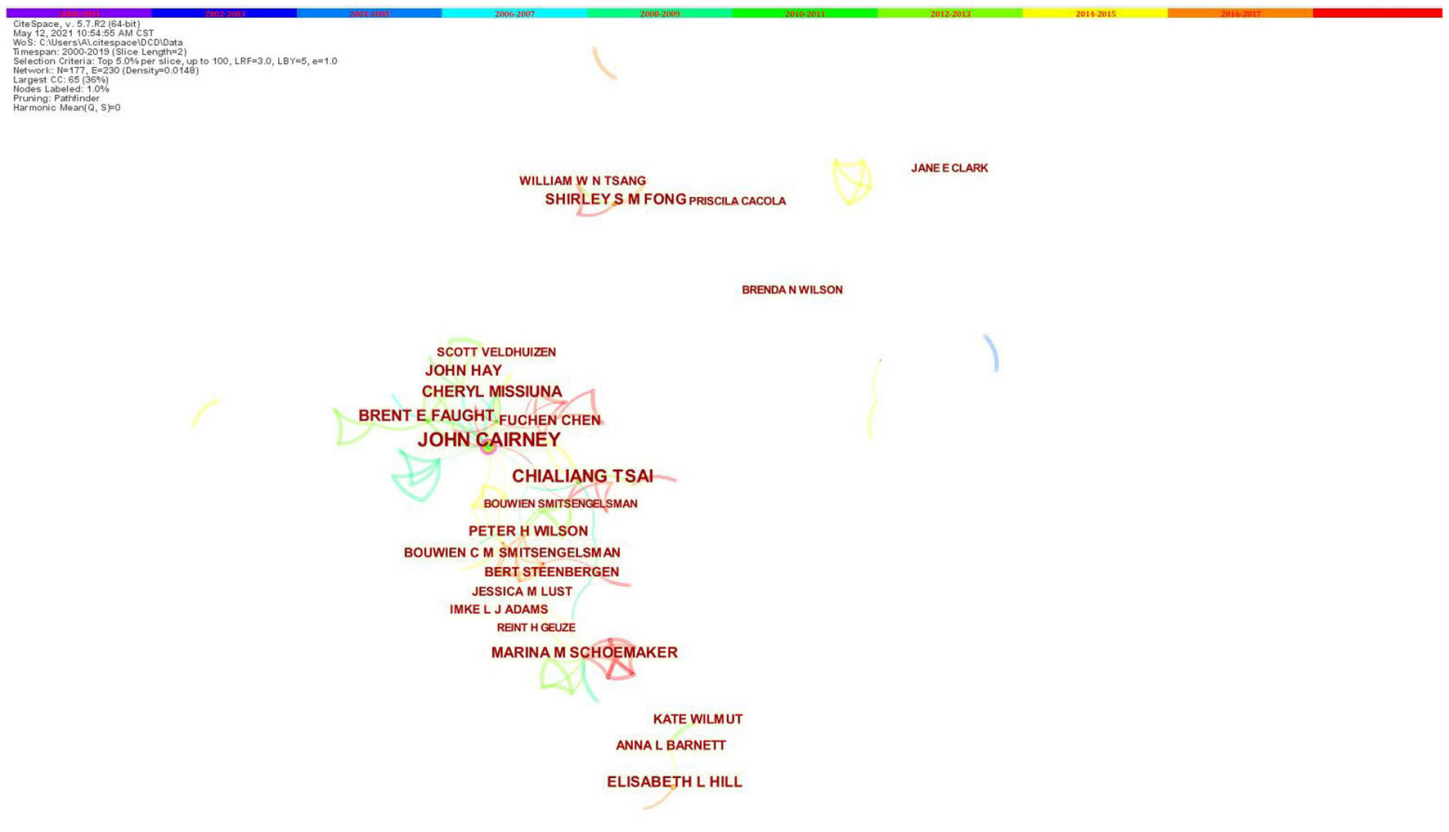
Co-authorship network map in research of children with DCD, 2000–2019. The bigger the circle, the more original articles the author published. The shorter and thicker the connection line, the closer relationship between authors.

Figure 4 is the co-authorship network map generated by CiteSpace. Regarding the authors who were active, John Cairney from the Department of Family Medicine, McMaster University, Canada, ranks the first (40 publications), followed by Chialiang Tsai from the Institute of Physical Education, Health and Leisure Studies, National Cheng Kung University, Taiwan (22 publications). The top 10 authors are presented in Table 3.

**Table 3:** Top 10 active authors in research of children with DCD, 2000–2019.

**Ranking**	**Author**	**Publications**	**Centrality**
1	John Cairney	40	0.16
2	Chialiang Tsai	22	0.04
3	Brent E Faught	18	0.03
4	Cheryl Missiuna	17	0.07
5	Elisabeth L. Hill	16	0.00
6	John Hay	14	0.03
7	Shirley S. M. Fong	13	0.00
8	Marina M. Schoemaker	13	0.06
9	Peter H. Wilson	12	0.05
10	Fuchen Chen	11	0.02

### Research Topic Analysis

#### Reference Co-citation

**Figure 5:**
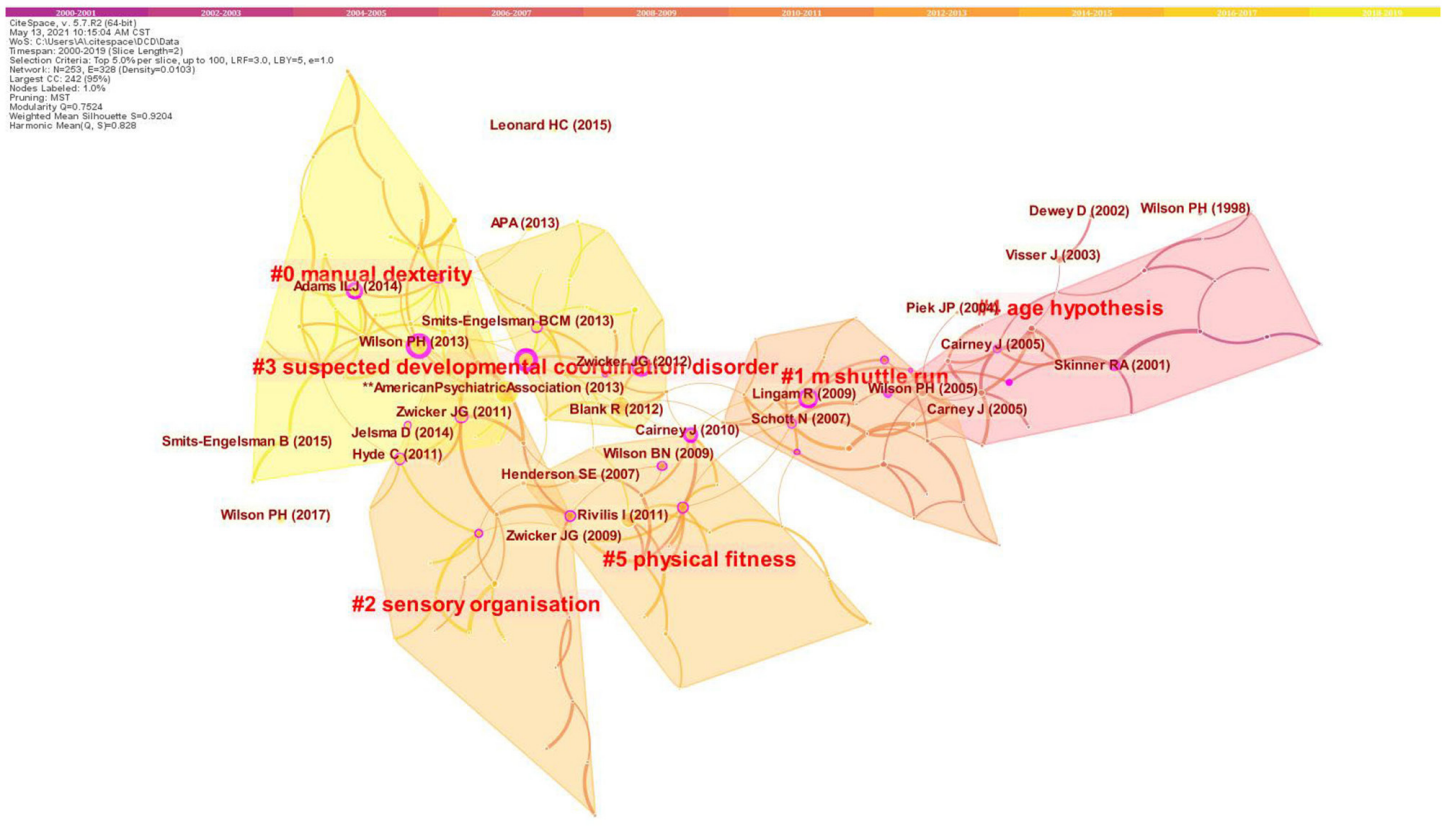
Clustering map of reference co-citation related to research of children with DCD, 2000–2019. The larger the circle, the more frequently it is co-cited. The thicker the purple circle, the stronger the betweenness centrality.

After selecting reference as node type for statistical analysis, it shows that 635 original records containing 12,559 references were primary downloaded and entered the database. Colors, moving from purple to yellow, indicate the citation time from 2000 to 2019, respectively. The clusters are named by extracting nominal terms as labels from the titles of the cited articles. The LLR (log-likelihood ratio) algorithm is used as the extraction method. Figure 5 shows a cluster visualization of the reference co-citation network which is divided into six co-citation clusters including 0# manual dexterity, 1# m shuttle run, 2# sensory organization, 3# suspected DCD, 4# age hypothesis, and 5# physical fitness. The summary of the largest five clusters is shown in Table 4.

**Table 4:** Summary of the largest five clusters.

**Cluster ID**	**Size**	**Silhouette**	**Label (LLR)**	**Mean (cite year)**	**Description**
0	32	0.942	Manual dexterity	2013	An important manifestation of developmental coordination disorder
1	24	0.889	M shuttle run	2007	A well-established field measure of maximal oxygen uptake in children
2	24	0.967	Sensory organization	2009	Children with developmental coordination disorder have widespread impairment in their sensory organization
3	21	0.857	Suspected developmental coordination disorder	2011	A group of people exposed to risk factors for developmental coordination disorder
4	21	0.878	Age hypothesis	2002	The activity-deficit between children with and without coordination problems widens with age

*Size: the number of references that a cluster contains*.

*LLR, log-likelihood ratio*.

The top ranked article by citation counts (Table 5) is American Psychiatric Association (15) in Cluster #0, with citation counts of 111. The second one is Blank et al. (16) in Cluster #3, with citation counts of 85. The third is Wilson et al. (17) in Cluster #0, with citation counts of 75. The fourth is Zwicker et al. (3) in Cluster #3, with citation counts of 44.

**Table 5:** Top five co-citation references related to children with DCD, 2000–2019.

**Ranking**	**Frequency**	**Centrality**	**Source**	**Cited reference**	**References**	**Cluster**
1	111	0.09	DIAGN STAT MAN MENT	American Psychiatric Association (2013). Diagnostic and Statistical Manual of Mental Disorders. 5th Edition, APA, Washington, DC	(15)	#0
2	85	0.08	DEV MED CHILD NEUROL	European Academy for Childhood Disability (EACD): Recommendations on the definition, diagnosis and intervention of developmental coordination disorder (long version)	(16)	#3
3	75	0.48	DEV MED CHILD NEUROL	Understanding performance deficits in developmental coordination disorder: a meta-analysis of recent research	(17)	#0
4	44	0.31	EUR J PAEDIATR NEURO	Developmental coordination disorder: A review and update	(3)	#3
5	42	0.06	RES DEV DISABIL	Physical activity and fitness in children with developmental coordination disorder: A systematic review	(18)	#5

### Journal/Instruction Manual Co-citation

A total of 12,559 citation references are from different journals/instruction manuals. Table 6 lists the top 10 highly cited journals/instruction manuals. The highest cited journal is the Developmental Medicine and Child Neurology, with 548 citations, followed by the Journal of Human Movement Science (547) and Movement Assessment Battery for Children (462).

**Table 6:** Top 10 highly cited journals in research of children with DCD, 2000–2019.

**Ranking**	**Journal**	**Frequency**	**Centrality**	**Impact factor (2019)**
1	Developmental Medicine and Child Neurology	548	0.16	4.406
2	Human Movement Science	547	0.27	2.096
3	Movement Assessment Battery for Children^*^	462	0.07	—
4	Diagnostic and Statistical Manual of Mental Disorder^*^	460	0.07	—
5	Research in Developmental Disabilities	353	0.23	1.836
6	Child: Care, Health & Development	320	0.14	1.918
7	Adapted Physical Activity Quarterly	305	0.24	1.462
8	Journal of Child Psychology and Psychiatry	255	0.04	6.129
9	Pediatrics	216	0.05	5.401
10	American Journal of Occupational Therapy	206	0.14	1.952

* *These two are manuals*.

### Future Research Direction Analysis

**Figure 6:**
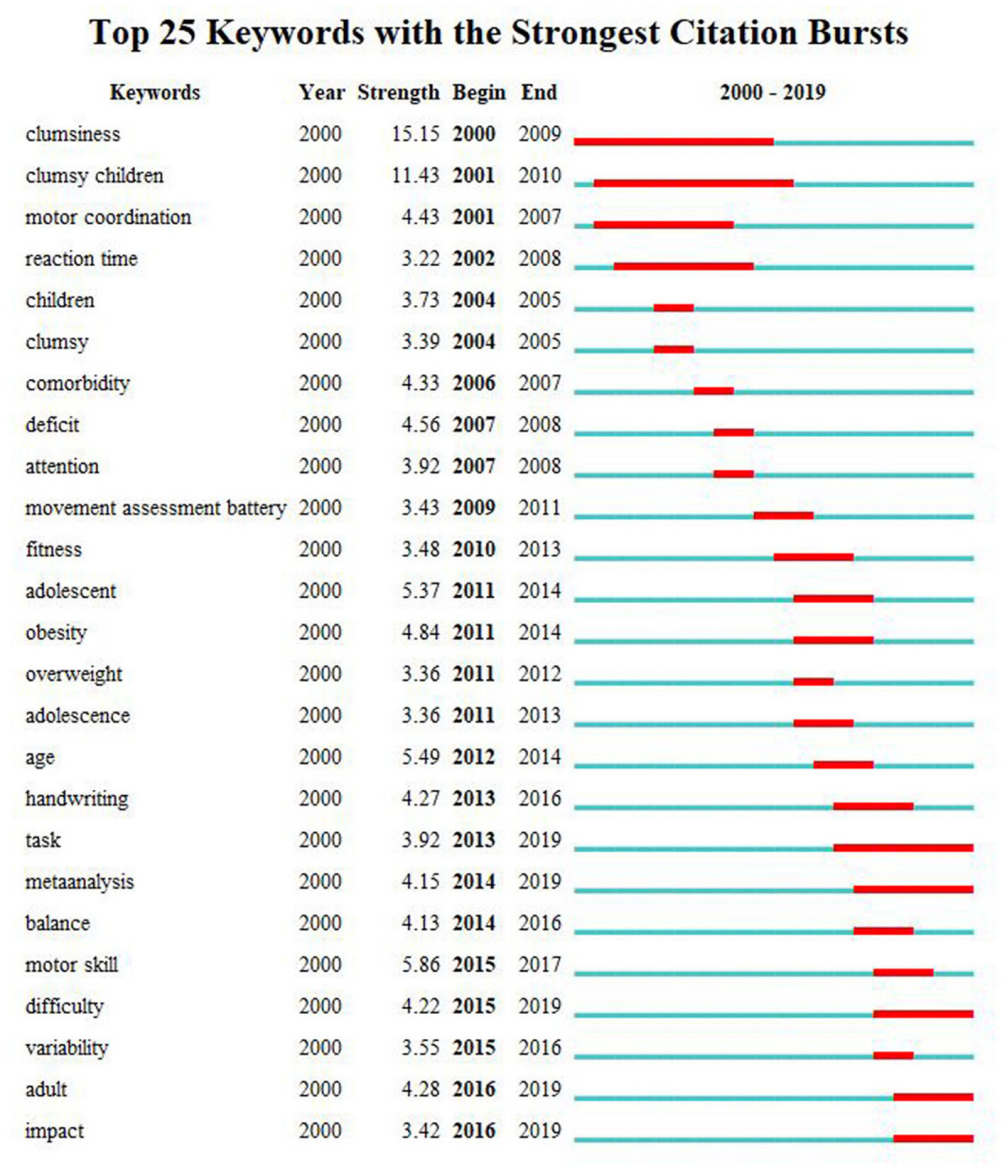
Keywords with the strongest citation bursts in published articles on children with DCD, 2000–2019. The timeline is depicted as a blue line, and the time interval that a subject was found to have a burst is shown as a red segment which indicated the beginning year, the ending year, and the duration of the burst.

The burst detection in CiteSpace is based on Kleinberg's algorithm, which based on modeling the stream using an infinite-state automaton to extract a meaningful structure from document streams that arrive continuously over time (19). These analyses can show the fast-growing topics that last for multiple years as well as a single year. Figure 6 shows the top 25 keywords with the strongest citation bursts in published articles on children with DCD. The blue line represents the time interval, and the red line refers to the duration of the citation burst. In the keyword's citation burst detection analysis, clumsiness is the strongest burst keyword that appeared in 2000 with the burst strength of 15.15, followed by clumsy children (11.43), motor skill (5.86), and age (5.49). Five frontiers in the field of children with DCD that have impacts on future research are task, meta-analysis, difficulty, adult, and impact.

## Discussion

### Quantity and Cooperation Analysis

In this study, we utilized information visualization to analyze original articles on children with DCD published from 2000 through 2019. Before the expert meeting 1994, a wide variation in terminology and diagnostic criteria was used to describe children with DCD, hampering the external validity of the scoping review. In 2001, a survey of 234 professionals from the UK, including doctors, occupational therapists, and speech therapists, found that the term “dyspraxia” is much more widely used in the UK rather than DCD (20). So, in 2012, the European Academy for Childhood Disability (EACD) published the recommendations on the definition, diagnosis, and intervention of DCD (16). Unified nomenclature is very critical and the most basic requirement for the diagnosis and treatment of a clinical disease in the later stage. In this study, we identified an increasing number of scientific research publications over the last 20-year period. However, it can be seen from Figure 1 that the quantity of papers published before 2005 was still very low.

In this article, Canada's contribution to this field is the most outstanding, accounting for 21.57% (137/635) of the articles in this field in the past 20 years. By analyzing the nodes of authors, cooperative relationship with others could be investigated. Dr. Cairney the Director of the Infant and Child Health Research Laboratory at both the University of Toronto and McMaster University (21) (currently Head of the School of Human Movement and Nutrition Sciences at University of Queensland in Australia), is the most productive researcher in this field. His research examined the relationships among motor skill, physical activity, and physical and mental health in children (22). He is internationally known for his work on DCD and its impact on physical and psychological well-being in children (with the strongest centrality of 0.16). McMaster University also becomes the most productive (frequency: 54) and influential (centrality: 0.74) research institution in this field. Besides, it is obvious that the collaboration in investigating children with DCD is relatively close, mostly linked with institutions with high centrality, such as McMaster University, University of Toronto, and University of Cape Town. The top two institutions both come from Canada, indicating the leading role in this field.

### Research Topic

In a co-citation network, references cited by a given article provide valuable information regarding intellectual connections between various scientific concepts (12). The literature represented in the co-citation network is organized into six different clusters. Each node represents a cited article, and the clusters represent a distinct specialty or a thematic concentration. The silhouette value of a cluster measures the quality of a clustering configuration, which ranges between −1 and 1. The higher the silhouette score (recommendation value >0.70), the more consistent of the cluster members are, providing the clusters in comparison have similar sizes (23).

During the past two decades, a significant amount of research has been conducted to study children with DCD. Compromised *manual dexterity* is a hallmark of the DCD symptom profile (24, 25), which has become a focus point of children with DCD research during more recent years. *Manual Dexterity* has also become one of the three components of the Movement Assessment Battery for Children, Second Edition (MABC-2), which is a validated, standardized, and norm-referenced test used to measure motor proficiency in children with DCD (26).

Besides *manual dexterity* problems, children with DCD are also known to have lower levels of physical fitness, including cardiorespiratory fitness, anaerobic capacity, and muscle strength. Sprinting tests are commonly used to assess general anaerobic capacity, but only the *shuttle run* item of the Bruininks–Oseretsky Test of Motor Proficiency, Second Edition (BOT-2), and the 10 × 5 m sprint were investigated, which are currently the best choices (27, 28).

Actually, balance dysfunction is one of the most common sensorimotor impairments observed among children with DCD. However, until now, a comprehensive understanding of the etiology is still lacking. A majority of studies suggested that the impaired postural control can be ascribed to a deficit in central control and *sensory organization* (29). The *sensory organization* test (SOT), which has demonstrated good reliability and validity, was used to evaluate the *sensory organization* of balance control for children with DCD (30, 31).

*Age hypothesis* was presented that the activity deficit in children with DCD would grow larger as children's play became more complex and rule-bound (32). In other words, problems and secondary consequences in early childhood can persist through childhood and into adolescence and adulthood in children with DCD (33). To prevent these problems, early recognition of *suspected DCD* seems to be paramount in order to give the child the necessary support.

Therefore, many kinds of questionnaires have been designed to detect the play characteristics of young children who are suspected for DCD. The DCDQ-R (Developmental Coordination Disorder Questionnaire, Revised Version) has been shown to be one of the most utilized screening tools for DCD and a useful adjunct in studies using clinical samples (34–36). Further, the DCDQ-R has been revised for use in younger children that are 3 and 4 years of age: the Little DCD Questionnaire or Little DCDQ (18).

### Research Fundamental

The top co-cited articles are often considered fundamental and a basis for a certain research field. Based on co-citation networks, top-cited publications are further analyzed to investigate the knowledge base for the field of children with DCD. Therefore, by combining with Figure 5 (thick purple ring) and Table 5 (centrality: 0.48 and 0.31), we find that high-quality meta-analysis published in the *DEV MED CHILD NEUROL* by Wilson et al. (17) and literature review published in the *EUR J PAEDIATR NEURO* by Zwicker et al. (3) are the solid foundation of the research in this field. Meta-analysis was also widely used in systematic reviews in many other disciplines and fields. Especially, it plays an important role in the field of evidence-based medicine and public health (37). Among the listed top five co-cited references in Table 5, the first two are both guidelines/manuals in this field (15, 16), which play fundamental and instructive roles in the research of children with DCD. These two articles belong to cluster #0 and #3, respectively, which labeled “manual dexterity” and “suspected developmental coordination disorder.”

### Research Source

The highly cited journals reflect the best source in the research field of children with DCD to a certain extent. *Developmental Medicine* & *Child Neurology* has defined the field of pediatric neurology and childhood-onset neurodisability. It is a multidisciplinary journal, one of the world's leading journals in the whole field of pediatrics. As a kind of specific motor skill developmental disorder, the assessment of DCD is very critical. Therefore, main studies should refer to the assessment tool or manual, such as the MABC-1/2, and the Diagnostic and Statistical Manual of Mental Disorder, Fifth Edition (DSM-V).

### Future Research Direction

From Figure 6, the hot topics switched from *clumsiness*, the burst keyword with the most strength, *clumsy children, motor coordination* to *movement assessment battery, adolescent*, and finally to *task, meta-analysis, difficulty, adult, impact*, and so on. Research foci in children with DCD seems to have shifted from children to adolescent and adult, from research in general symptom to specific assessment. Moreover, research foci mainly focused on clinical research rather than basic science or mechanism. Based on the evolution of keywords bursts and the ending year displayed with red segment, the future research trends in this field could be concluded as follows.

①DCD is a neurodevelopmental abnormality found in healthy children and persisting in “*adulthood*,” which may “*impact*” everyday living (38). Therefore, new studies were conducted for “*adolescents/adults*” in the latest recommendations in 2019 to update the recommendations in 2012 (35). In recent years, many case–control and longitudinal studies have been reported (39, 40), and it will become one of the hot spots in this field in the future.

②Since motor control and executive function deficits are expressed as a function of “*task type*” and difficulty, interventions for DCD can be broadly categorized into two types: process or deficit-oriented and “*task-specific*” (3, 35). “*Task-specific*” training was found to be useful in improving the performance of children with DCD based on the type of task trained (41, 42). Some reviews and meta-analysis also found that approaches from a task-specific perspective yielded stronger effects, whereas this preliminary evidence is currently insufficient (43, 44). Therefore, it will be also one of the research trends in this field.

### Strengths and Limitations

To our knowledge, this bibliometric study is the first of its kind to identify and characterize research in children with DCD. Moreover, our findings provide a clear visual analysis, from the quantity, quality, citation analysis, and so on, of the publications of children with DCD. It will be a good example of visualization analysis available to explore research hotspots in other fields.

There are also some weaknesses to the study. Although we used the WoSCC for our bibliometric analysis, there are other public and commercially available bibliometric databases, such as Scopus, Medline, and PubMed. However, there are some reasons for this choice of database. For example, PubMed records do not include information on cited references so it is not possible to perform citation analysis using PubMed (45).

## Conclusion

From the current bibliometric analysis of children with DCD publications over the previous 20 years, it is found that related publications increased at a rapid rate. Canada has the highest publication rate and centrality, and the institution and researcher that are most prolific in this field are also from Canada. The analysis and interpretation of the research literature will be very helpful to the clinicians and scientists in this field.

## Author Contributions

M-QW, D-QW, C-PH, and L-SI contributed to study design and acquisition of research data. M-QW conducted the data analysis. M-QW and D-QW drafted the manuscript. All authors contributed to improvements of the manuscript for important intellectual content and approved the final version for publication.

## Funding

This study was supported by Shanghai Pudong Municipal Health Commission (PW2020D-11), the Science and Technology Commission of Shanghai Municipality (19140903100), Shanghai Municipal Health Commission (2020YJZX0213), and Clinical Research Plan of Shanghai Hospital Development Center (SHDC2020CR1047B-003). The funders had no role in the conduct of the study, the analysis or interpretation of data, and the preparation, review, or approval of the manuscript.

## Conflict of Interest

The authors declare that the research was conducted in the absence of any commercial or financial relationships that could be construed as a potential conflict of interest.

## Publisher's Note

All claims expressed in this article are solely those of the authors and do not necessarily represent those of their affiliated organizations, or those of the publisher, the editors and the reviewers. Any product that may be evaluated in this article, or claim that may be made by its manufacturer, is not guaranteed or endorsed by the publisher.

## Abbreviations

BOT-2: Bruininks–Oseretsky Test of Motor Proficiency Second Edition
DCD: developmental coordination disorder
DSM-V: Diagnostic and Statistical Manual of Mental Disorder Fifth Edition
EACD: European Academy for Childhood Disability
LLR: log-likelihood ratio
MABC-2: Movement Assessment Battery for Children Second Edition
SOT: sensory organization test
WoSCC: Web of Science Core Collection.

## References

[1] van Hoorn JF Schoemaker MM Stuive I Dijkstra PU Rodrigues Trigo Pereira F van der Sluis CK. Risk factors in early life for developmental coordination disorder: a scoping review. Dev Med Child Neurol. (2021) 63:511–9. 10.1111/dmcn.14781 33345317 PMC8048603

[2] Zwicker JG Lee EJ. Early intervention for children with/at risk of developmental coordination disorder: a scoping review. Dev Med Child Neurol. (2021) 63:659–67. 10.1111/dmcn.14804 33426644

[3] Zwicker JG Missiuna C Harris SR Boyd LA. Developmental coordination disorder: a review and update. Eur J Paediatr Neurol. (2012) 16:573–81. 10.1016/j.ejpn.2012.05.005 22705270

[4] Mandich A Polatajko HJ. Developmental coordination disorder: mechanisms, measurement and management. Hum Mov Sci. (2003) 22:407–11. 10.1016/j.humov.2003.09.001 14624825

[5] Polatajko H Fox M Missiuna C. An international consensus on children with developmental coordination disorder. Can J Occup Ther. (1995) 62:3–6. 10.1177/00084174950620010130671947

[6] Brandt JS Hadaya O Schuster M Rosen T Sauer MV Ananth CV. bibliometric analysis of top-cited journal articles in obstetrics and gynecology. JAMA Netw Open. (2019) 2:e1918007. 10.1001/jamanetworkopen.2019.18007 31860106 PMC6991228

[7] Yang L He L Ma Y Wu L Zhang Z. A visualized investigation on the intellectual structure and evolution of waste printed circuit board research during 2000-2016. Environ Sci Pollut Res Int. (2019) 26:11336–41. 10.1007/s11356-019-04590-8 30798494

[8] Zhang D Xu JP Zhang YZ Wang J He SY Zhou X. Study on sustainable urbanization literature based on web of science, scopus, and china national knowledge infrastructure: a scientometric analysis in citespace. J Clean Prod. (2020) 264:121537. 10.1016/j.jclepro.2020.121537

[9] Azam A Ahmed A Wang H Wang YE Zhang ZT. Knowledge structure and research progress in wind power generation (wpg) from 2005 to 2020 using citespace based scientometric analysis. J Clean Prod. (2021) 295:126496. 10.1016/j.jclepro.2021.126496

[10] Chen C Hu Z Liu S Tseng H. Emerging trends in regenerative medicine: a scientometric analysis in citespace. Expert Opin Biol Ther. (2012) 12:593–608. 10.1517/14712598.2012.674507 22443895

[11] Takahashi K Yamanaka S. Induction of pluripotent stem cells from mouse embryonic and adult fibroblast cultures by defined factors. Cell. (2006) 126:663–76. 10.1016/j.cell.2006.07.024 16904174

[12] Chen C Dubin R Kim MC. Emerging trends and new developments in regenerative medicine: a scientometric update (2000-2014). Expert Opin Biol Ther. (2014) 14:1295–317. 10.1517/14712598.2014.920813 25077605

[13] Yi F Yang P Sheng H. Tracing the scientific outputs in the field of ebola research based on publications in the web of science. BMC Res Notes. (2016) 9:221. 10.1186/s13104-016-2026-2 27083891 PMC4832479

[14] Chen C. Searching for intellectual turning points: progressive knowledge domain visualization. Proc Natl Acad Sci USA. (2004) 101:5303–10. 10.1073/pnas.0307513100 14724295 PMC387312

[15] Association AP. Diagnostic and statistical manual of mental disorders. 5th edition, Washington, DC. (2013).

[16] Blank R Smits-Engelsman B Polatajko H Wilson P. European academy for childhood disability (eacd): recommendations on the definition, diagnosis and intervention of developmental coordination disorder (long version). Dev Med Child Neurol. (2012) 54:54–93. 10.1111/j.1469-8749.2011.04171.x 22171930

[17] Wilson PH Ruddock S Smits-Engelsman B Polatajko H Blank R. Understanding performance deficits in developmental coordination disorder: a meta-analysis of recent research. Dev Med Child Neurol. (2013) 55:217–28. 10.1111/j.1469-8749.2012.04436.x 23106668

[18] Rihtman T Wilson BN Parush S. Development of the little developmental coordination disorder questionnaire for preschoolers and preliminary evidence of its psychometric properties in israel. Res Dev Disabil. (2011) 32:1378–87. 10.1016/j.ridd.2010.12.040 21295440

[19] Kleinberg J. Bursty and hierarchical structure in streams. Data Min Knowl Discov. (2003) 7:373–97. 10.1023/A:1024940629314

[20] Peters JM Barnett AL Henderson SE. Clumsiness, dyspraxia and developmental co-ordination disorder: How do health and educational professionals in the uk define the terms? Child Care Health Dev. (2001) 27:399–412. 10.1046/j.1365-2214.2001.00217.x 11531913

[21] Cairney J Veldhuizen S Rodriguez MC King-Dowling S Kwan MY Wade T. Cohort profile: the canadian coordination and activity tracking in children (catch) longitudinal cohort. BMJ Open. (2019) 9:e029784. 10.1136/bmjopen-2019-029784 31501117 PMC6738750

[22] Professor john cairney 2021.

[23] Li Y Fang R Liu Z Jiang L Zhang J Li H. The association between toxic pesticide environmental exposure and alzheimer's disease: a scientometric and visualization analysis. Chemosphere. (2021) 263:128238. 10.1016/j.chemosphere.2020.128238 33297185

[24] Fuelscher I Caeyenberghs K Enticott PG Williams J Lum J Hyde C. Differential activation of brain areas in children with developmental coordination disorder during tasks of manual dexterity: an ale meta-analysis. Neurosci Biobehav Rev. (2018) 86:77–84. 10.1016/j.neubiorev.2018.01.002 29339004

[25] Nobusako S Sakai A Tsujimoto T Shuto T Nishi Y Asano D. Deficits in visuo-motor temporal integration impacts manual dexterity in probable developmental coordination disorder. Front Neurol. (2018) 9:114. 10.3389/fneur.2018.00114 29556211 PMC5844924

[26] Bonney E Aertssen W Smits-Engelsman B. Psychometric properties of field-based anaerobic capacity tests in children with developmental coordination disorder. Disabil Rehabil. (2019) 41:1803–14. 10.1080/09638288.2018.1446189 29509037

[27] Aertssen W Jelsma D Smits-Engelsman B. Field-based tests of strength and anaerobic capacity used in children with developmental coordination disorder: a systematic review. Phys Ther. (2020) 100:1825–51. 10.1093/ptj/pzaa118 32949239

[28] Cairney J Hay J Veldhuizen S Faught B. Comparison of vo2 maximum obtained from 20 m shuttle run and cycle ergometer in children with and without developmental coordination disorder. Res Dev Disabil. (2010) 31:1332–9. 10.1016/j.ridd.2010.07.008 20702060

[29] Speedtsberg MB Christensen SB Andersen KK Bencke J Jensen BR Curtis DJ. Impaired postural control in children with developmental coordination disorder is related to less efficient central as well as peripheral control. Gait Posture. (2017) 51:1–6. 10.1016/j.gaitpost.2016.09.019 27693806

[30] Fong SS Lee VY Pang MY. Sensory organization of balance control in children with developmental coordination disorder. Res Dev Disabil. (2011) 32:2376–82. 10.1016/j.ridd.2011.07.025 21835590

[31] Fong SS Tsang WW Ng GY. Taekwondo training improves sensory organization and balance control in children with developmental coordination disorder: a randomized controlled trial. Res Dev Disabil. (2012) 33:85–95. 10.1016/j.ridd.2011.08.023 22093652

[32] John C Hay J Faught BE Corna LM Flouris AD. Developmental coordination disorder, age and play: a test of the divergence in activity-deficit with age hypothesis. Adapt Phys Act Q. (2006) 23:261–76. 10.1123/apaq.23.3.261

[33] Cantell M Houwen S Schoemaker M. Age-related validity and reliability of the dutch little developmental coordination disorder questionnaire (ldcdq-nl). Res Dev Disabil. (2019) 84:28–35. 10.1016/j.ridd.2018.02.010 29477487

[34] Wilson BN Crawford SG Green D Roberts G Aylott A Kaplan BJ. Psychometric properties of the revised developmental coordination disorder questionnaire. Phys Occup Ther Pediatr. (2009) 29:182–202. 10.1080/01942630902784761 19401931

[35] Blank R Barnett AL Cairney J Green D Kirby A Polatajko H. International clinical practice recommendations on the definition, diagnosis, assessment, intervention, and psychosocial aspects of developmental coordination disorder. Dev Med Child Neurol. (2019) 61:242–85. 10.1111/dmcn.14132 30671947 PMC6850610

[36] Patel P Gabbard C. Adaptation and preliminary testing of the developmental coordination disorder questionnaire (dcdq) for children in india. Phys Occup Ther Pediatr. (2017) 37:170–82. 10.3109/01942638.2016.1150383 27058012

[37] Zhu C Jiang T Cao H Sun W Chen Z Liu J. Longitudinal analysis of meta-analysis literatures in the database of isi web of science. Int J Clin Exp Med. (2015) 8:3559–65. 26064249 PMC4443083

[38] Farmer M Echenne B Drouin R Bentourkia M. Insights in developmental coordination disorder. Curr Pediatr Rev. (2017) 13:111–9. 10.2174/1573396313666170726113550 28745216

[39] Landgren V Fernell E Gillberg C Landgren M Johnson M. Attention-deficit/hyperactivity disorder with developmental coordination disorder: 24-year follow-up of a population-based sample. BMC Psychiatry. (2021) 21:161. 10.1186/s12888-021-03154-w 33752617 PMC7983399

[40] Warlop G Vansteenkiste P Lenoir M Deconinck FJA. Young adults with developmental coordination disorder adopt a different visual strategy during a hazard perception test for cyclists. Front Psychol. (2021) 12:665189. 10.3389/fpsyg.2021.665189 33935926 PMC8079720

[41] Fong SS Guo X Liu KP Ki WY Louie LH Chung RC. Task-specific balance training improves the sensory organisation of balance control in children with developmental coordination disorder: a randomised controlled trial. Sci Rep. (2016) 6:20945. 10.1038/srep20945 26864309 PMC4750073

[42] Cavalcante Neto JL Steenbergen B Zamunér AR Tudella E. Wii training versus non-wii task-specific training on motor learning in children with developmental coordination disorder: a randomized controlled trial. Ann Phys Rehabil Med. (2021) 64:101390. 10.1016/j.rehab.2020.03.013 32445975

[43] Smits-Engelsman BC Blank R van der Kaay AC Mosterd-van der Meijs R Vlugt-van den Brand E Polatajko HJ. Efficacy of interventions to improve motor performance in children with developmental coordination disorder: A combined systematic review and meta-analysis. Dev Med Child Neurol. (2013) 55:229–37. 10.1111/dmcn.12008 23106530

[44] Miyahara M Hillier SL Pridham L Nakagawa S. Task-oriented interventions for children with developmental co-ordination disorder. Cochrane Database Syst Rev. (2017) 7:Cd010914. 10.1002/14651858.CD010914.pub2 28758189 PMC6483344

[45] Wu D Wang S Hu C Yan C Wu M. Ten years of the cohort biobank: bibliometric outcomes. Biopreserv Biobank. (2021) 19:269–79. 10.1089/bio.2020.0096 33449812

